# Management and Prognosis of Interstitial Lung Disease With Lung Cancer (ILD-LC): A Real-World Cohort From Three Medical Centers in China

**DOI:** 10.3389/fmolb.2021.660800

**Published:** 2021-03-31

**Authors:** Xie Xiaohong, Wang Liqiang, Li Na, Lin Xinqing, Qin Yinyin, Liu Ming, Ouyang Ming, Han Qian, Luo Qun, Li Shiyue, Li Chunyan, Wang Xiaoqian, Yang Shuanying, Huang Wei, Liu Mei, Wang Ping, Zhou Chengzhi

**Affiliations:** ^1^State Key Laboratory of Respiratory Disease, National Clinical Research Center of Respiratory Disease, The First Affiliated Hospital of Guangzhou Medical University, Guangzhou Institute of the Respiratory Health, Guangzhou Medical University, Guangzhou, China; ^2^Department of Respiratory and Critical Care Medicine, The Second Affiliated Hospital of Xi’an Jiaotong University, Xi’an, China; ^3^Department of Respiratory and Critical Care Medicine, The First Medical Center of PLA General Hospital, Beijing, China; ^4^Department of Respiratory and Critical Care Medicine, The Eighth Medical Center of PLA General Hospital, Beijing, China

**Keywords:** interstitial lung disease, lung cancer, ILD-LC, prognosis, anti-angiogenesis treatment

## Abstract

**Background and Objective:**

Interstitial lung disease with lung cancer (ILD-LC) is rare and its management has not been fully described. This study aimed to investigate the management and prognosis of ILD-LC patients in China.

**Methods:**

The present analysis is a retrospective real-world cohort study. Clinical data of ILD-LC patients were obtained from 3 hospitals in China. The overall survival (OS) of patients was analyzed. Univariate and multivariate regression analyses were performed.

**Results:**

One hundred eighty-four ILD-LC patients included were biased toward male (85.3%), smokers (75.5%), idiopathic pulmonary fibrosis (IPF) (58.2%) patients with comorbidities (67.9%) and ECOG-PS score of 1 (65.2%). Most patients were advanced peripheral non-small cell lung cancer. The initial anti-cancer regimen for ILD-LC is mainly chemotherapy, and patients with early-stage LC prefer surgery. In the anti-cancer cohort, the number of ILD-LC patients who underwent the 2nd and 3rd or more anti-cancer regimens were 78 (55.7%) and 32 (22.8%), respectively. In the non-anticancer cohort, the median OS was 3.5 months. In the early-stage cohort, the median OS was 14.2 months in the systematic therapy group; however, the median OS was not reached in the surgery group. In the advanced-stage cohort with systematic therapy, the median OS was 7.2 months. Interstitial pneumonia (IIP) and anti-angiogenesis were associated with OS in the univariate analysis, whereas anti-angiogenesis was an independent protective factor for advanced LC with ILD.

**Conclusion:**

Patients with ILD-LC have very poor prognosis. Appropriate anti-tumor treatment can prolong the survival time of patients who can tolerate it. Targeted therapy and immunotherapy are alternative treatments for LC patients with mild ILD. For ILD patients with advanced LC, antiangiogenic regimens significantly improve the prognosis of the disease.

## Introduction

Interstitial lung disease (ILD), especially idiopathic pulmonary fibrosis (IPF) and connective tissue disease (CTD)-ILD, is a risk factor for lung cancer. The incidence of lung cancer is 4.4–48% in IPF patients, while 5.5–9.0% in CTD-ILD patients (Raghu et al., 2004; Tomassetti et al., 2015; Enomoto et al., 2016; Jung et al., 2018; Watanabe et al., 2018). Risk factors for developing ILD with lung cancer (ILD-LC) include male sex, older age, smoking, combined pulmonary fibrosis and emphysema (CPFE) (Usui et al., 2011; Watanabe et al., 2018). In addition, CTD-ILD without immunosuppressive therapy may be a risk factor for cancer (Watanabe et al., 2018).

Recently, the clinical profile of patients with ILD-LC was described. The ILD-LC population comprises individuals who are male, elderly, smokers, have IPF and are at advanced stage of LC (Minegishi et al., 2009). High-resolution computed tomography (HRCT) shows that the tumors are mainly in peripheral areas around ILD (Minegishi et al., 2009; Yoo et al., 2019). Squamous cell carcinoma was considered to be the most common pathological type of ILD-LC (Tomassetti et al., 2015; Watanabe et al., 2018), but there was an increasing trend for adenocarcinoma and small cell carcinoma nowadays (Saijo et al., 2017).

The IPF guidelines recommend that IPF be considered in treatment decisions for LC in IPF-LC patients (Cottin et al., 2017; Homma et al., 2018). However, the LC guidelines have not yet been established for the treatment of these patients. Surgery is recommended for early-stage lung cancer patients with ILD (Homma et al., 2018); however, there is still controversy over whether active anti-tumor strategies are suitable for inoperable ILD-LC patients with pulmonary dysfunction and poor PS score. The acute exacerbation (AE) of ILD (AE-ILD) is the main reason for the difficulty in treating patients with anti-cancer therapy (Minegishi et al.,2011a,b; Sekine et al., 2016; Hanibuchi et al., 2018; Asahina et al., 2019; Fujimoto et al., 2019; Kenmotsu et al., 2019). Although there are a few prospective studies involving chemotherapy and immunotherapy (Minegishi et al., 2009; Naccache et al., 2018), the current researches on inoperable ILD-LC focus on describing the treatments and AE-ILD retrospectively in a small population. Reducing AE-ILD is urgent, and several studies have shown that anti-angiogenic drugs such as nintedanib and bevacizumab can reduce the risk of chemotherapy-related AE-ILD (Richeldi et al., 2014; Hamada et al., 2019). However, there are more anti-angiogenic drugs, such as apatinib, anlotinib and recombinant human endostatin, etc., which have an unknown effect in reducing AE-ILD.

To date, there is no large-scale research on the treatment and prognosis of ILD-LC in China. Besides, the treatment of lung cancer has undergone tremendous changes, so the management of ILD-LC needs to be further described. To better understand the current management and prognosis of ILD-LC in China, we conducted a retrospective study to provide a feasible reference for the follow-up treatment for ILD-LC in real clinical practice.

## Patients and Methods

### Patients and Diagnostic Criteria

A systematic search was conducted in the databases of three medical centers, The First Affiliated Hospital of Guangzhou Medical University, Chinese People’s Liberation Army General Hospital, and The Second Affiliated Hospital of Xi’an Jiaotong University, located in Southeast, North, and Northwest China. All included cases presented with ILD on chest HRCT scan and were histologically diagnosed with LC from January 1, 2009 to October 1, 2019. Those with missing clinical data, other malignant tumors, drug-related ILD, and cancerous lymphangitis were excluded.

Patients eligible were grouped into idiopathic interstitial pneumonia (IIP) and CTD-ILD groups based on HRCT findings and clinical courses. We classify IIP into two groups: the IPF pattern and non-IPF groups. The IPF pattern group consisted of patients diagnosed as IPF histologically or clinically according to the American Thoracic Society/European Respiratory Society (ATS/ERS) criteria. IPF diagnosed without pathology is mainly based on clinical manifestations and typical chest CT findings: basal and peripheral predominant reticular abnormalities with traction bronchiectasis and honeycombing (ATS and ERS, 2002).

### Data Collection

Data such as demographic characteristics, as well as information on smoking history, comorbidities, the subtype of ILD, pulmonary function test, time to diagnose LC, histology according to World Health Organization (WHO) tumor classification, stage group using the 8th edition of the TNM classification, and regimens for anti-cancer, was obtained retrospectively from the medical record. Two independent pulmonologists evaluated the chest HRCT images taken during the diagnosis of ILD-LC that met the criteria. The imaging evaluation mainly distinguishes the IPF pattern which is mentioned above. The location of the mass is recorded as either peripheral or central. The peripheral location is defined as <3 cm from the pleura. The disagreement between the 2 reviewers has been resolved by consensus.

### Statistical Analysis

SPSS software version 24 (IBM) was used for statistical analysis and Graphpad Prism version 8 for graphing. Patient demographics were compared using the unpaired t-test for continuous variables and Pearson’s χ^2^ test for categorical variables. Overall survival (OS) was defined as the time from the date of diagnosis of primary lung cancer to the date of death from any cause. Patients who were alive or had been lost to follow-up at the time of analysis were considered censored. Kaplan-Meier was used for survival analysis and the log-rank test was used to assess differences between groups. Cox’s proportional hazards regression analysis was used to identify significant variables that affect the survival of advanced ILD-LC patients and to estimate hazard ratios (HR) and 95% confidence intervals (CI) for predictors of survival. All tests were two-tailed, and *p* < 0.05 was considered statistically significant.

## Results

### Patient Characteristics

**FIGURE 1:**
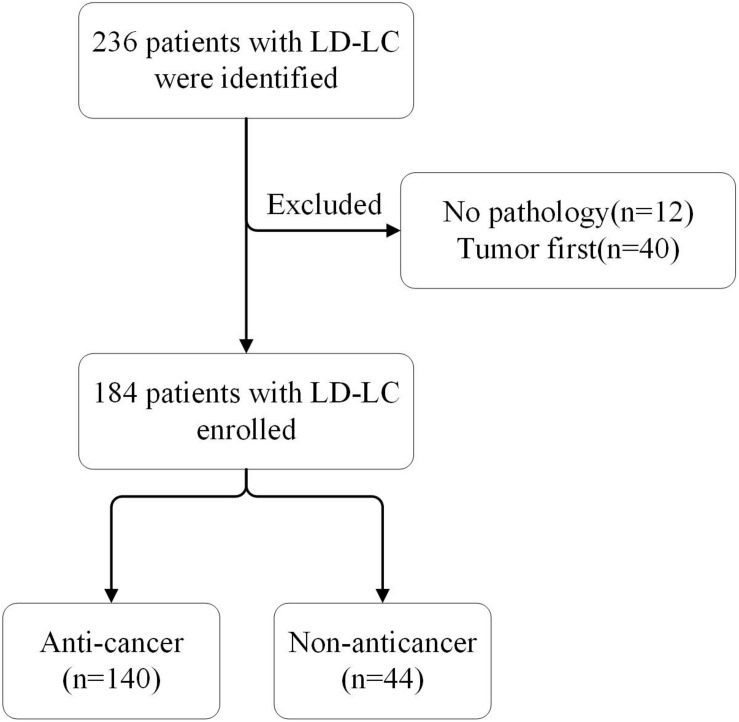
Flowchart of the ILD-LC patients selecting process.

A total of 236 patients with ILD-LC were identified, and 184 were eventually enrolled in the study (Figure 1). The baseline characteristics of all patients, the anti-cancer group and the non-anticancer group are shown in Table 1. Overall, at the time of LC diagnosis, the majority of the patients were male (85.3%) and smokers (75.5%) with a median age of 68 years (range, 44–90 years). The main subtype of ILD was IPF (58.2%). Most patients were stage III/IV peripheral NSCLC with comorbidities (67.9%) and ECOG-PS scores of 1 (65.2%). There were differences in age, ECOG-PS, and smoking status between the anti-cancer and non-anticancer groups. Patients in the non-anticancer group had older age and worse ECOG-PS scores.

**TABLE 1:** Characteristics of anti-cancer and non-anticancer patients with interstitial lung disease and lung cancer (ILD-LC).

Characteristics	All patients (*N* = 184)	Anti-cancer (*N* = 140)	Non-anticancer (*N* = 44)	*p-*value
Gender, male, *n* (%)	157 (85.3)	121 (86.4)	36 (81.8)	0.451
Age, median (range), year	68.0 (44–90)	66.0 (44–90)	70.5 (46–89)	0.005
ECOG-PS				0.001
1	120 (65.2)	99 (70.7)	21 (47.7)	
2	57 (31.0)	39 (27.9)	18 (40.9)	
3	7 (3.8)	2 (1.4)	5 (11.4)	
Smoking status, current or former, *n* (%)	139 (75.5)	111 (79.2)	28 (63.6)	0.035
Smoking index	885 ± 514	881 ± 500	901 ± 577	0.850
Comorbidities, yes, *n* (%)	125(67.9)	93 (66.4)	32 (72.7)	0.435
Histology				0.36
Squamous cell carcinoma	58 (31.5)	42 (30.0)	16 (36.4)	
Adenocarcinoma	69 (37.5)	50 (35.7)	19 (43.2)	
Other NSCLC	17 (9.2)	15 (10.7)	2 (4.5)	
Small cell carcinoma	40 (21.7)	33 (23.6)	7 (15.9)	
Clinical stage				0.536
I	19 (10.3)	17 (12.1)	2 (4.5)	
II	12 (6.5)	9 (6.4)	3 (6.8)	
III	52 (28.3)	38 (27.1)	14 (31.8)	
IV	101 (54.9)	76 (54.3)	25 (56.8)	
Anatomical type				0.776
Peripheral	145 (78.8)	111 (79.3)	34 (77.3)	
Central	39 (21.2)	29 (20.7)	10 (22.7)	
Subtype of ILD				0.107
IPF	107 (58.2)	78 (55.7)	29 (65.9)	
non-IPF	56 (30.4)	48 (34.3)	8 (18.2)	
CTD-ILD	21 (11.4)	14 (10.0)	7 (15.9)	
Time to diagnose LC				0.781
After ILD	87 (47.3)	67 (47.9)	20 (45.5)	
Simultaneous	97(52.7)	73 (52.1)	24 (54.5)	
Disorder of ventilation function (*n*)	68	47	21	0.071
Normal	15 (22.1)	14 (29.8)	1 (4.8)	
Restrictive	32 (47.1)	20 (42.6)	12 (57.1)	
Mixed	21 (30.9)	13 (27.6)	8 (38.1)	
Degree of ventilation function (*n*)	68	47	21	0.521
Normal or Mild	42 (61.8)	31 (66.0)	11 (52.4)	
Moderate	17 (25.0)	10 (21.3)	7 (33.3)	
Severe	9 (13.2)	6 (12.8)	3 (14.3)	
Degree of diffusion capacity (*n*)	61	43	18	0.077
Normal or mild	24 (39.3)	20 (46.5)	4 (22.2)	
Moderate and severe	37 (60.7)	23 (53.5)	14 (77.8)	

**ECOG-PS, Eastern Cooperative Oncology Group-Performance Status; ILD, interstitial lung disease; IPF, idiopathic pulmonary fibrosis; CTD, connective tissue disease; NSCLC, non-small cell lung cancer; LC, lung cancer. The p-values refer to comparisons between the anti-cancer and non-anti-cancer groups.**

### The Distribution of Various Anti-cancer Regimens at Different Treatment Stages in ILD-LC

Most patients with ILD-LC could not undergo multiple courses of anti-cancer regimens (Table 2). Compared with the initial treatment, the number of patients underwent the 2nd and 3rd or more anti-cancer regimens were 78 (55.7%) and 32 (22.8%), respectively. Chemotherapy accounted for the largest proportion of all treatment stages. In addition to chemotherapy, surgery (17.9%) and radiotherapy (28.2%) were the most common in the first-stage treatment and the second- stage treatment, respectively. Besides, anti-angiogenic therapy (25.0%) is preferred in the third and above stage therapies.

**TABLE 2:** Initial and follow-up anti-cancer regimens for patients with ILD-LC.

Stage of treatment^#^	Totol	1st- (*n*,%^&^)	2nd- (*n*,%)	≥3rd-or more (*n*,%)
*N*	140	140 (100.0)	78 (55.7)	32 (22.8)
Surgery	25	25 (17.9)	0 (0)	0 (0)
Chemotherapy	109	89 (63.6)	45 (57.7)	23 (71.9)
Interventional therapy	6	2 (1.4)	3 (3.8)	1 (3.1)
Radiotherapy	35	10 (7.1)	22 (28.2)	3 (9.4)
Anti-angiogenic therapy^※^	29	13 (9.3)	10 (12.8)	8 (25.0)
Targeted therapy^€^	20	12 (8.6)	8 (10.3)	3 (9.4)
Immunotherapy	8	5 (3.6)	3 (3.8)	1 (3.1)
Others^€^	7	7 (5.0)	0 (0)	0 (0)

**^#^Patients can receive more than one anti-cancer regimens at every stage of treatment. ^&^The proportion of a regimen in the treatment stage to total events of the regimen. ^※^Anti-angiogenic agents include 11 cases of bevacizumab, 3 cases of apatinib, 5 cases of anlotinib, 9 cases of nintedanib, 1 case of recombinant human endostatin. ^€^There were 11 patients with EGFR mutations,10 in the first stage and 1 in the second stage. Two patients in the first stage received EGFR-TKIs without confirming the EGFR status. The remaining patients in the second- and above stage were at an unknown EGFR status for lacking records. ^€^Oral anti-cancer drugs.**

### The Choice of Anti-cancer Regimens Based on Histological Type, Clinical-Stage, the Subtype of ILD, and ECOG-PS Score

The anti-cancer regimen for ILD-LC is similar to that for lung cancer alone (Table 3). Patients who underwent surgery were early-stage non-small cell lung cancer patients with good ECOG-PS scores and non-IPF can be found for a higher proportion of these patients. Patients with good ECOG-PS score, small cell lung cancer, advanced stage lung cancer, and non-IPF are more inclined to choose chemotherapy. Radiotherapy is mainly used for stage III/IV squamous cell carcinoma and small cell carcinoma with an ECOG-PS score of 1, and IPF accounts for more. Targeted therapy is mainly used for IIP patients with an ECOG-PS score of 2–3 and stage III/IV adenocarcinoma. Immune checkpoint inhibitors have also been used in our ILD-LC patients, who are characterized by advanced IIP-LC with better ECOG-PS scores.

**TABLE 3:** Treatment options for the anti-cancer cohort of ILD-LC stratified according to histological type, clinical stage, subtype of ILD, and ECOG-PS score.

	*N*	Surgery^&^	Chemotherapy	Interventional therapy	Radiotherapy	Anti-angiogenic therapy	Targeted therapy	Immunotherapy	Others
**Histology**									
Squamous cell carcinoma^#^	42	10 (23.8)	31 (73.8)	3 (7.1)	16 (38.1)	6 (14.3)	2 (4.7)	4 (9.5)	5 (11.9)
Adenocarcinoma	50	8 (16.0)	36 (72.0)	0 (0)	5 (10.0)	14 (28.0)	15 (30.0)	1 (2.0)	1 (2.0)
Other NSCLC	15	4 (26.7)	11 (73.3)	1 (6.7)	2 (13.3)	3 (20.0)	3 (20.0)	1 (6.7)	1 (6.7)
Small cell carcinoma	33	3 (9.1)	31 (93.9)	2 (6.1)	12 (36.4)	6 (18.2)	0 (0)	2 (6.1)	0 (0)
**Clinical stage**									
I	17	14 (82.4)	10 (58.8)	0 (0)	5 (29.4)	3 (17.6)	1 (5.9)	1 (5.9)	2 (11.8)
II	9	5 (55.6)	8 (88.9)	0 (0)	2 (22.2)	1 (11.1)	0 (0)	0 (0)	0 (0)
III	38	6 (15.8)	30 (78.9)	1 (2.6)	13 (34.2)	8 (21.1)	4 (10.5)	2 (5.3)	3 (7.9)
IV	76	0 (0)	61 (80.3)	5 (6.6)	15 (19.7)	17 (22.4)	15 (19.7)	5 (6.6)	2 (2.6)
**ILD classification**									
IPF	78	12 (15.4)	60 (76.9)	3 (3.8)	15 (19.2)	17 (21.8)	13 (16.7)	4 (5.1)	6 (7.7)
non-IPF	48	11 (22.9)	40 (83.3)	3 (6.3)	16 (33.3)	6 (12.5)	6 (12.5)	4 (8.3)	1 (2.1)
CTD-ILD	14	2 (14.3)	9 (64.3)	0 (0)	4 (28.6)	6 (42.9)	1 (7.1)	0 (0)	0 (0)
**ECOG-PS**									
1	99	23 (23.2)	81 (81.8)	2 (2.0)	35 (35.3)	21 (21.2)	8 (8.1)	7 (7.1)	6 (6.1)
2	39	2 (5.1)	27 (69.2)	3 (7.7)	0 (0)	7 (17.9)	12 (30.8)	1 (2.6)	1 (2.6)
3	2	0 (0)	0 (0)	1 (50)	0 (0)	1 (50)	0 (0)	0 (0)	0 (0)

**ECOG-PS, Eastern Cooperative Oncology Group-Performance Status; ILD, interstitial lung disease; IPF, idiopathic pulmonary fibrosis; CTD, connective tissue disease; NSCLC, non-small cell lung cancer. ^#^Patients in each stratification may receive one or more treatments. ^&^The proportion of a regimen in each stratification to the total number of the stratification.**

### Survival Data and Prognostic Factors

**FIGURE 2:**
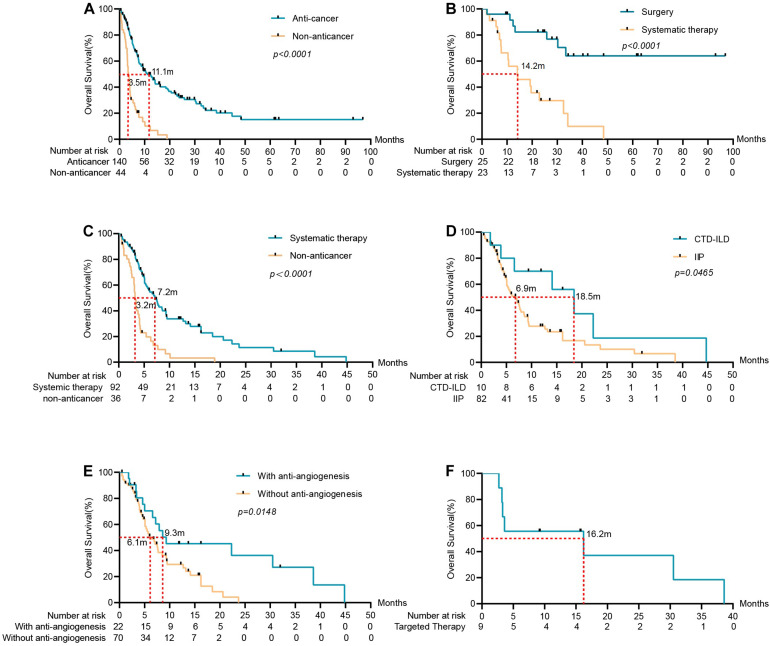
Survival analysis of ILD-LC patients in different groups. **(A)** Anti-cancer group (*n* = 140) vs. non-anticancer group (*n* = 44), the mOS of the anti-cancer group was significantly longer than that of the non-anticancer group. **(B)** Surgery group (*n* = 25) and systematic therapy group (*n* = 23) in early-stage LC with ILD. **(C)** Systematic therapy group (*n* = 92) vs. non-anticancer group in advanced stage LC with ILD. **(D)** For patients in advanced stage LC with ILD, OS was associated with the subtype of ILD (*p* = 0.0465). **(E)** OS was associated with anti-angiogenesis (*p* = 0.0148). **(F)** Patients received targeted therapy in advanced stage LC with ILD and the mOS was 16.2 months.

Patients were followed-up until May 2020. Thirty-four of the one hundred eighty-four patients were lost to follow-up and 23 were still alive. The median follow-up time was 27 months. In the overall population, the median OS for anti-cancer cohort and non-anticancer cohort were 11.1 vs. 3.5 (HR 0.3014, 95%CI: 0.1771 to 0.5130). In the non-anticancer cohort, the 6-month and 1-year OS rates were 25 and 6.8%, respectively. Furthermore, we divided the patients in the anti-cancer cohort into two groups: the early-stage (stage I-IIIA) group (*n* = 48) and the advanced stage (stage IIIB-IV) cohort (*n* = 92). In the early-stage group, 25 patients received surgery and 23 received systematic therapy. The advanced stage group all received systematic therapy. Among the surgical patients, 7 cases died, 13 cases survived, and 5 cases were lost to follow-up. The median OS was not reached for patients who underwent surgery, and the 1, 2, and 3-year OS rates were 80.0, 68.0, and 36.0%, respectively. The median OS for the early-stage group that underwent systematic therapy was 14.2 m, the 1 and 2-year OS rate was 47.8%, and 17.4%, respectively. The median OS of the advanced-stage cohort that underwent systematic therapy was 7.2 m, and the 1-year OS rate was 20.7% (Figure 2). Sixteen advanced-stage LC patients were receiving targeted therapy with a median OS of 6.5 months, while 9 of them with EGFR mutations receiving targeted therapy in the first stage have a median OS of 16.2 months. The median OS of 6 patients with advanced-stage LC receiving immunotherapy was 8.8 months.

Analysis of prognostic factors was conducted to identify significant variables that affect the OS of the advanced stage group (Table 4). Gender, age, smoking status, anatomical type, histological type, stage, the subtype of ILD, the sequence of tumor occurrence, and anti-cancer regimens were included in the study. Univariate analysis revealed that patients with CTD-ILD and antiangiogenic therapy had a significantly better prognosis. Anti-angiogenic therapy was the independent factor in the Cox regression model. Patients with anti-angiogenic therapy have significantly longer OS compared to those without anti-angiogenesis, with a median OS of 9.3 vs. 6.1 m (HR: 0.5002, 95% CI: 0.2866 to 0.8731, *p* = 0.0148).

**TABLE 4:** Clinical characteristics, univariate and multivariate analysis of 92 advanced lung cancer with ILD for overall survival.

		Overall survival	
Group	No. patients (%)	Univariate analysis: P	Multivariate analysis: P
Gender (male, %)	77 (83.7)	0.058	0.065
Age (≥65, %)	58 (63.0)	0.071	0.163
Smoking status (yes, %)	72 (78.3)	0.47	*
Comorbidities (yes, %)	64 (69.6)	0.103	0.113
Histology (NSCLC, %)	68 (73.9)	0.647	*
Clinical stage (IV, %)	72 (78.3)	0.323	*
Anatomical type (peripheral, %)	70 (76.1)	0.659	*
ECOG-PS (1, %)	62 (67.4)	0.551	*
Subtype of ILD (IIP, %)	82 (89.1)	**0.046**	0.138
Time to diagnose LC (simultaneous, %)	52 (56.5)	0.17	0.122
Interventional therapy (yes, %)	3 (3.3)	*	*
Radiotherapy (yes, %)	21 (22.8)	0.16	0.133
Chemotherapy (yes, %)	74 (80.4)	0.659	*
Anti-angiogenic therapy (yes, %)	22 (23.9)	**0.015**	**0.011**
Targeted therapy (yes, %)	16 (17.4)	0.736	*
Immunotherapy (yes, %)	6 (6.5)	*	*
Others (yes, %)	5 (5.4)	*	*

ECOG-PS, Eastern Cooperative Oncology Group-Performance Status; ILD, interstitial lung disease; CTD, connective tissue disease; NSCLC, non-small cell lung cancer; IIP, idiopathic interstitial pneumonia, including idiopathic pulmonary fibrosis (IPF) and non-IPF. *Analysis could not be done. The bold values means statistically different.

## Discussion

The current descriptions of the treatment and prognosis of ILD-LC are mainly derived from retrospective studies involving a small population, which are insufficient and limited. Here, we conducted a multicenter retrospective study involving 184 ILD-LC patients, with the aim of understanding the clinical profile, management, and prognosis of ILD-LC patients in China. Consistent with previous studies (Minegishi et al., 2009; Tomassetti et al., 2015), patients with ILD-LC in our cohort are mainly elderly, smokers, male. They also had IIP, peripheral NSCLC, poor ECOG-PS, and comorbidities. Half of the patients were diagnosed with lung cancer and ILD at the same time, and most of them had mild to moderate restrictive ventilatory dysfunction and moderate to severe diffuse dysfunction.

The management of ILD-LC is important and necessary but has not been fully described. The published articles hardly discuss the differences in choosing anti-cancer or not in patients with ILD-LC. In our study, 44 (23.9%) of 184 patients chose the best supportive care (BSC) without anti-cancer regimens. We found that older age, worse ECOG-PS scores, and poor lung function may be reasons for patients choosing the BSC. Furthermore, the anti-cancer regimens in our cohort are similar to that of patients with lung cancer only. However, the challenge in clinical practice is that AE induced by anti-cancer regimens may interrupt the treatment of LC and even cause the death of patients (Minegishi et al., 2009; Amundson et al., 2019). We did not summarize the AE-ILD in most patients for the lack of clinical data. Therefore, the number of patients who underwent anti-cancer treatment at different stages was used to reflect the tolerance of these patients to anti-cancer treatment. There were 55.7% of patients administered second- and above stage treatments in our study, while 36.3% (32 of 88 in anticancer) in Minegishi et al. (2009). However, only 22.8% of ILD-LC patients underwent a third or more anti-cancer treatment in the anti-cancer group. All this means that many patients have lost the opportunity to receive more anti-cancer treatments because of ILD.

Appropriate anti-cancer measures for ILD-LC are reasonable, which makes patients live longer. Surgery is important and recommended for the management of early-stage LC with ILD due to the high survival rate (Omori et al., 2015; Sato et al., 2015; Homma et al., 2018). We also concluded that the 3-year OS rates of surgery cohort were 36.0%. However, postoperative AE-ILD is a serious complication for patients with surgery. There was one patient who experienced postoperative AE-ILD and died within 2 months in our study. Sato et al. (2014) showed that 9.3% of 1,763 patients suffered postoperative AE-ILD with a mortality rate of 43.9%. Minegishi et al. (2009) reported that 8 of 35 patients who underwent surgery experienced postoperative AE-ILD, and 3 died of AE. Surgical procedures, male sex, history of exacerbation, preoperative steroid use, serum sialylated carbohydrate antigen KL-6 levels, preoperative percent vital capacity plus serum LDH values were risk factors for postoperative AE-ILD after in patients with ILD (Shintani et al., 2010; Sato et al., 2014).

Systematic therapy for inoperable or recurrent LC with ILD is still controversial due to the AE-ILD. As mentioned above, most patients fail to receive multi-stage anti-cancer treatments in our study. However, consistent with the published researches (Kawahara et al., 2019), the present study showed that patients with advanced-stage LC benefit from systematic therapy in OS compared to those without anti-cancer treatment. The options of systematic treatment are diverse, and there is no optimal treatment for ILD-LC. Consistent with the previous study, chemotherapy was the most commonly chosen in our patients. However, the chemotherapy regimen for less AE is still inconclusive and chemotherapy-related exacerbation of ILD was between 9 and 42.8% (Watanabe et al., 2013; Kenmotsu et al., 2015). A series of prospective studies conducted in Japan showed that nab-paclitaxel plus carboplatin in NSCLC and etoposide plus carboplatin in SCLC were safe for patients with mild or moderate ILD (Minegishi et al.,2011a,b; Kenmotsu et al., 2019).

Radiotherapy plays a critical role in unresectable SCLC and NSCLC but should be used with caution in ILD-LC due to severe radiation pneumonia or AE-ILD (Yamaguchi et al., 2013; Glick et al., 2018). There were 35 patients with mild ILD administered with radiotherapy in our study, and 2 of them suffered grade 3 radiation pneumonia and AE-ILD respectively. The patient with radiation pneumonia received nintedanib monotherapy for more than a year and is still alive. While the patient with AE-ILD received BSC and died with an OS of 8 months. Radiotherapy-related grade 2–5 pneumonitis of patients with ILD-LC was 15.8% (Yamaguchi et al., 2013). Hagiwara et al. (2020) conducted a nationwide survey of radiotherapy in Japan for ILD-LC and found that 5(7.5%) of 67 patients suffered AE-ILD and 1 died of radiation-induced AE. Kobayashi et al. (2018) revealed that 17 (46%) of 37 patients treated with chemoradiotherapy developed grade 3 and above AE-ILD within 1 year after the last irradiation. Therefore, radiotherapy should be applied cautiously considering the serious side effects.

For patients with driver genes and mutations, tyrosine kinase inhibitors (TKIs) are more effective than traditional chemotherapy. However, the risk of TKI-induced ILD and AE-ILD remind us that TKIs should be applied cautiously in ILD-LC (Shi et al., 2014; Shah, 2016; Gemma et al., 2019). In our cohort, there were 10 patients confirmed with EGFR mutations receiving EGFR-TKIs in first-line treatment. The median OS of the 9 patients with advanced-stage LC was 16.2 months, and 4 (44.4%) of them experienced more than one stage treatments. Besides, preliminary evidence suggests that TKIs can be continued after TKI-induced ILD in the case of steroid coverage and/or dose reduction (Shah, 2016). Considering the long-term survival of ILD-LC patients with driver genes and mutations, TKIs should be administered at the first stage treatments.

The application of immune checkpoint inhibitors (ICIs) is a milestone in lung cancer, and the occurrence of immune-related adverse events (irAEs) such as checkpoint inhibitor-related pneumonitis (CIPs) is inevitable (Jain et al., 2018). The high mortality rate was observed in severe grade CIPs in NSCLC (Suresh et al., 2018; Tone et al., 2019), and pre-existing ILD is a risk factor for developing CIPs (Kanai et al., 2018). Therefore, CIPs maybe a serious threat to patients with ILD-LC though there was no report of AE-ILD induced by ICIs. However, compared with TKIs-related pneumonia, CIPs have a higher response rate to corticosteroid therapy and a lower mortality rate (Beom et al., 2016; Kato et al., 2017). For inoperable patients with mild IIP receiving nivolumab, a pilot study conducted by Fujimoto et al. (2017, 2019)showed no AE-ILD, while a phase II showed that 2 of 18 patients developed grade 2 pneumonitis, which could be treated using corticosteroid therapy. There was no AE-ILD or CIP observed in our advanced stage LC patients receiving immunotherapy with a median OS of 8.6 months, while 1 of them died of tumor progression within 1 month. This suggests that immunotherapy may be a good choice for these patients, but more clinical studies are needed to confirm it.

For advanced LC with ILD, the most appropriate anti-cancer regimens should bring the fewest AEs rather than the best anti-tumor effect. However, it is still necessary to look for prognostic factors that affect OS. We conducted a prognosis analysis in the advanced anti-cancer cohort and found the IIP was associated with poor prognosis, while anti-angiogenic therapy was an independent protective factor that improves the prognosis of advanced LC with ILD. There was in line with expectations that IIP is a prognostic risk factor of ILD-LC, because IIP, especially IPF, has a worse prognosis compared to CTD-ILD (Lim et al., 2019). Vascular endothelial growth factor (VEGF), a potent stimulator of vascular permeability and angiogenesis, plays an important role in lung diseases, including injury, fibrosis and cancer, as well as AE of IPF (Simler et al., 2004; Weis and Cheresh, 2005; McKeown et al., 2009). Nintedanib, an anti-angiogenic agent targeting VEGF, has been proven to have anti-cancer and anti-pulmonary fibrosis effects and reduce AE-ILD in patients with IPF (Reck et al., 2014; Richeldi et al., 2014; Hilberg et al., 2018; Fabre and Nicholson, 2020). Besides, nintedanib and bevacizumab have been shown to reduce the risk of chemotherapy-related AE-ILD in patients with lung cancer (Shimizu et al., 2014; Enomoto et al., 2015; Hamada et al., 2019). Anlotinib, a new anti-angiogenesis agent approved globally in 2018, has been proven attenuating bleomycin-induced pulmonary fibrosis in mice via the TGF-β1 signaling pathway (Ruan et al., 2020). Recombinant human endostatin and apatinib have not been evaluated to reduce AE in patients with ILD. In our study, anti-angiogenic agents mentioned above were included in the analysis. Although we failed to confirm whether anti-angiogenesis can reduce the acute exacerbation of ILD, we found that patients with advanced-stage LC receiving systematic therapy benefited from anti-angiogenic therapy. The possible reasons for anti-angiogenic therapy as an independent protective factor are as follows: 1. exerting the anti-tumor effect directly; 2. reducing AE-ILD and enabling patients to receive more anti-tumor treatments. Therefore, anti-angiogenic agents may be a good choice for patients with ILD-LC.

ILD-LC is a rare disease with poor prognosis, which is a part of severe lung cancer (Xie et al., 2019). These patients often face challenges in anti-cancer treatment because of poor PS scores or lung function caused by the progression of ILD. Furthermore, they may fail to receive anti-cancer treatment due to the slow progression or treatment-related AE-ILD. However, our research shows that patients with ILD-LC benefit from anti-cancer treatment. Our research shows that patients with ILD-LC benefit from anti-cancer treatment. In particular, these patients can live longer after using anti-angiogenic drugs, which suggests that anti-angiogenic therapy may be an important part of ILD-LC anti-cancer therapy.

Several limitations need to be noted regarding this real-world study. First, this is a retrospective study conducted over a long time. This study failed to reflect the current treatment status of ILD-LC due to the anti-cancer therapy of lung cancer has undergone great changes. Second, the lack of acute exacerbation and lung function data in most patients makes it impossible for us to analyze the toxicity and true efficacy of anti-cancer treatments. Our interpretation of survival data is inadequate. Third, the present study involves a variety of anti-angiogenic drugs and cannot be analyzed separately due to the limited sample size. Further investigations and larger patient groups are needed to confirm the protective effect of anti-angiogenic agents in patients with ILD-LC treated with anti-cancer therapy.

## Conclusion

Patients with ILD-LC have very poor prognosis. Appropriate anti-tumor treatment can prolong the survival time of patients who can tolerate it. Targeted therapy and immunotherapy are alternative treatments for LC patients with mild ILD. For ILD patients with advanced LC, anti-angiogenic regimens significantly improve their prognosis.

## Data Availability Statement

The raw data supporting the conclusions of this article will be made available by the authors, without undue reservation.

## Ethics Statement

The studies involving human participants were reviewed and approved by the Institutional Review Board of The First Affiliated Hospital of Guangzhou Medical University (Guangzhou, Guangdong, China). Written informed consent for participation was not required for this study in accordance with the national legislation and the institutional requirements.

## Author Contributions

XX, WL, and ZC: conception and design. QY, LMi, OM, HQ, LQ, LS, LC, WX, YS, HW, LMe, WP, and ZC: provision of study materials or patients. LW and LN: collection and assembly of data. XX, LW, LN, LX, LC, WX, YS, and HW: data analysis and interpretation. XX, WL, and LN: manuscript writing. All authors: final approval of the manuscript.

## Conflict of Interest

The authors declare that the research was conducted in the absence of any commercial or financial relationships that could be construed as a potential conflict of interest.

## References

[B1] Amundson W. H. Racila E. Allen T. Dincer H. E. Tomic R. (2019). Acute exacerbation of interstitial lung disease after procedures. *Respir. Med.* 150 30–37. 10.1016/j.rmed.2019.02.012 30961948

[B2] Asahina H. Oizumi S. Takamura K. Harada T. Harada M. (2019). A prospective phase II study of carboplatin and nab-paclitaxel in patients with advanced non-small cell lung cancer and concomitant interstitial lung disease (HOT1302). *Lung Cancer* 138 65–71. 10.1016/j.lungcan.2019.09.020 31654836

[B3] ATS and ERS (2002). American Thoracic Society/European Respiratory Society International Multidisciplinary Consensus Classification of the Idiopathic Interstitial Pneumonias. This joint statement of the American Thoracic Society (ATS), and the European Respiratory Society (ERS) was adopted by the ATS board of directors, June 2001 and by the ERS Executive Committee, June 2001. *Am. J. Respir. Crit. Care Med.* 165 277–304. 10.1164/ajrccm.165.2.ats01 11790668

[B4] Beom S. H. Kim D. W. Sim S. H. Keam B. Park J. H. (2016). Gefitinib-Induced interstitial lung disease in korean lung cancer patients. *Cancer Res. Treat.* 48 88–97. 10.4143/crt.2014.201 25761482 PMC4720097

[B5] Cottin V. Crestani B. Cadranel J. Cordier J. F. Marchand-Adam S. (2017). French practical guidelines for the diagnosis and management of idiopathic pulmonary fibrosis - 2017 update. Full-length version. *Rev. Mal. Respir.* 34 900–968. 10.1016/j.rmr.2017.07.017 28939155

[B6] Enomoto Y. Inui N. Yoshimura K. Nishimoto K. Mori K. (2016). Lung cancer development in patients with connective tissue disease-related interstitial lung disease: A retrospective observational study. *Medicine* 95:e5716. 10.1097/MD.0000000000005716 27977621 PMC5268067

[B7] Enomoto Y. Kenmotsu H. Watanabe N. Baba T. Murakami H. (2015). Efficacy and safety of combined carboplatin, paclitaxel, and bevacizumab for patients with advanced non-squamous non-small cell lung cancer with pre-existing interstitial lung disease: A retrospective multi-institutional study. *Anticancer Res.* 35 4259–4263. 26124387

[B8] Fabre A. Nicholson A. G. (2020). Nintedanib in progressive fibrosing interstitial lung diseases. *N. Engl. J. Med.* 382:780. 10.1056/NEJMc1917224 32074431

[B9] Fujimoto D. Morimoto T. Ito J. Sato Y. Ito M. (2017). A pilot trial of nivolumab treatment for advanced non-small cell lung cancer patients with mild idiopathic interstitial pneumonia. *Lung Cancer* 111 1–5. 10.1016/j.lungcan.2017.06.008 28838377

[B10] Fujimoto D. Yomota M. Sekine A. Morita M. Morimoto T. (2019). Nivolumab for advanced non-small cell lung cancer patients with mild idiopathic interstitial pneumonia: A multicenter, open-label single-arm phase II trial. *Lung Cancer* 134 274–278. 10.1016/j.lungcan.2019.06.001 31182249

[B11] Gemma A. Kusumoto M. Kurihara Y. Masuda N. Banno S. (2019). Interstitial lung disease onset and its risk factors in japanese patients with ALK-Positive NSCLC after treatment with crizotinib. *J. Thorac. Oncol.* 14 672–682. 10.1016/j.jtho.2018.11.022 30521972

[B12] Glick D. Lyen S. Kandel S. Shapera S. Le L. W. (2018). Impact of pretreatment interstitial lung disease on radiation pneumonitis and survival in patients treated with lung stereotactic body radiation therapy (SBRT). *Clin. Lung Cancer* 19 219–226. 10.1016/j.cllc.2017.06.021 29066051

[B13] Hagiwara Y. Nakayama Y. Kudo S. Hayakawa T. Nakamura N. (2020). Nationwide survey of radiation therapy in Japan for lung cancer complicated with interstitial lung disease. *J. Radiat. Res.* 61 563–574. 10.1093/jrr/rraa018 32363376 PMC7336568

[B14] Hamada S. Ichiyasu H. Ikeda T. Inaba M. Kashiwabara K. (2019). Protective effect of bevacizumab on chemotherapy-related acute exacerbation of interstitial lung disease in patients with advanced non-squamous non-small cell lung cancer. *BMC Pulmonary Med.* 19:72. 10.1186/s12890-019-0838-2 30940113 PMC6446385

[B15] Hanibuchi M. Kakiuchi S. Atagi S. Ogushi F. Shimizu E. (2018). A multicenter, open-label, phase II trial of S-1 plus carboplatin in advanced non-small cell lung cancer patients with interstitial lung disease. *Lung Cancer* 125 93–99. 10.1016/j.lungcan.2018.09.007 30429044

[B16] Hilberg F. Tontsch-Grunt U. Baum A. Le A. T. Doebele R. C. (2018). Triple angiokinase inhibitor nintedanib directly inhibits tumor cell growth and induces tumor shrinkage via blocking oncogenic receptor tyrosine kinases. *J. Pharmacol. Exp. Ther.* 364 494–503. 10.1124/jpet.117.244129 29263244 PMC6040086

[B17] Homma S. Bando M. Azuma A. Sakamoto S. Sugino K. (2018). Japanese guideline for the treatment of idiopathic pulmonary fibrosis. *Respir. Investig.* 56 268–291. 10.1016/j.resinv.2018.03.003 29980444

[B18] Jain A. Shannon V. R. Sheshadri A. (2018). Immune-Related adverse events: Pneumonitis. *Adv. Exp. Med. Biol.* 995 131–149. 10.1007/978-3-030-02505-2_6 30539509

[B19] Jung H. I. Park J. S. Lee M. Park B. Kim H. J. (2018). Prevalence of lung cancer in patients with interstitial lung disease is higher than in those with chronic obstructive pulmonary disease. *Medicine* 97:e71. 10.1097/MD.0000000000010071 29538197 PMC5882384

[B20] Kanai O. Kim Y. H. Demura Y. Kanai M. Ito T. (2018). Efficacy and safety of nivolumab in non-small cell lung cancer with preexisting interstitial lung disease. *Thorac. Cancer* 9 847–855. 10.1111/1759-7714.12759 29782069 PMC6026605

[B21] Kato T. Masuda N. Nakanishi Y. Takahashi M. Hida T. (2017). Nivolumab-induced interstitial lung disease analysis of two phase II studies patients with recurrent or advanced non-small-cell lung cancer. *Lung Cancer* 104 111–118. 10.1016/j.lungcan.2016.12.016 28212992

[B22] Kawahara T. Sakashita H. Suzuki T. Tateishi T. Miyazaki Y. (2019). Real world data of combined lung cancer and interstitial lung disease. *J. Thorac. Dis.* 11 4144–4151. 10.21037/jtd.2019.10.01 31737297 PMC6837971

[B23] Kenmotsu H. Naito T. Mori K. Ko R. Ono A. (2015). Effect of platinum-based chemotherapy for non-small cell lung cancer patients with interstitial lung disease. *Cancer Chemother. Pharmacol.* 75 521–526. 10.1007/s00280-014-2670-y 25563718

[B24] Kenmotsu H. Yoh K. Mori K. Ono A. Baba T. (2019). Phase II study of nab-paclitaxel + carboplatin for patients with non-small-cell lung cancer and interstitial lung disease. *Cancer Sci.* 110 3738–3745. 10.1111/cas.14217 31608537 PMC6890441

[B25] Kobayashi H. Naito T. Omae K. Omori S. Nakashima K. (2018). Impact of interstitial lung disease classification on the development of acute exacerbation of interstitial lung disease and prognosis in patients with stage III Non-Small-Cell lung cancer and interstitial lung disease treated with chemoradiotherapy. *J. Cancer* 9 2054–2060. 10.7150/jca.24936 29896291 PMC5995939

[B26] Lim J. U. Gil B. M. Kang H. S. Oh J. Kim Y. H. (2019). Interstitial pneumonia with autoimmune features show better survival and less exacerbations compared to idiopathic pulmonary fibrosis. *BMC Pulm. Med.* 19:120. 10.1186/s12890-019-0868-9 31272428 PMC6610995

[B27] McKeown S. Richter A. G. O’Kane C. McAuley D. F. Thickett D. R. (2009). MMP expression and abnormal lung permeability are important determinants of outcome in IPF. *Eur. Respir. J.* 33 77–84. 10.1183/09031936.00060708 18829682

[B28] Minegishi Y. Kuribayashi H. Kitamura K. Mizutani H. Kosaihira S. (2011a). The feasibility study of Carboplatin plus Etoposide for advanced small cell lung cancer with idiopathic interstitial pneumonias. *J. Thorac. Oncol.* 6 801–807. 10.1097/JTO.0b013e3182103d3c 21336181

[B29] Minegishi Y. Sudoh J. Kuribayasi H. Mizutani H. Seike M. (2011b). The safety and efficacy of weekly paclitaxel in combination with carboplatin for advanced non-small cell lung cancer with idiopathic interstitial pneumonias. *Lung Cancer* 71 70–74. 10.1016/j.lungcan.2010.04.014 20493578

[B30] Minegishi Y. Takenaka K. Mizutani H. Sudoh J. Noro R. (2009). Exacerbation of idiopathic interstitial pneumonias associated with lung cancer therapy. *Intern. Med.* 48 665–672. 10.2169/internalmedicine.48.1650 19420811

[B31] Naccache J. M. Gibiot Q. Monnet I. Antoine M. Wislez M. (2018). Lung cancer and interstitial lung disease: A literature review. *J. Thorac. Dis.* 10 3829–3844. 10.21037/jtd.2018.05.75 30069384 PMC6051867

[B32] Omori T. Tajiri M. Baba T. Ogura T. Iwasawa T. (2015). Pulmonary resection for lung cancer in patients with idiopathic interstitial pneumonia. *Ann. Thorac. Surg.* 100 954–960. 10.1016/j.athoracsur.2015.03.094 26116477

[B33] Raghu G. Nyberg F. Morgan G. (2004). The epidemiology of interstitial lung disease and its association with lung cancer. *Br. J. Cancer* 91(Suppl. 2), S3–S10. 10.1038/sj.bjc.6602061 15340372 PMC2750810

[B34] Reck M. Kaiser R. Mellemgaard A. Douillard J. Y. Orlov S. (2014). Docetaxel plus nintedanib versus docetaxel plus placebo in patients with previously treated non-small-cell lung cancer (LUME-Lung 1): A phase 3, double-blind, randomised controlled trial. *Lancet Oncol.* 15 143–155. 10.1016/S1470-2045(13)70586-2 24411639

[B35] Richeldi L. du Bois R. M. Raghu G. Azuma A. Brown K. K. (2014). Efficacy and safety of nintedanib in idiopathic pulmonary fibrosis. *N. Engl. J. Med.* 370 2071–2082. 10.1056/NEJMoa1402584 24836310

[B36] Ruan H. Lv Z. Liu S. Zhang L. Huang K. (2020). Anlotinib attenuated bleomycin-induced pulmonary fibrosis via the TGF-beta1 signalling pathway. *J. Pharm. Pharmacol.* 72 44–55. 10.1111/jphp.13183 31659758

[B37] Saijo A. Hanibuchi M. Goto H. Toyoda Y. Tezuka T. (2017). An analysis of the clinical features of lung cancer in patients with connective tissue diseases. *Respir. Investig.* 55 153–160. 10.1016/j.resinv.2016.11.003 28274531

[B38] Sato T. Teramukai S. Kondo H. Watanabe A. Ebina M. (2014). Impact and predictors of acute exacerbation of interstitial lung diseases after pulmonary resection for lung cancer. *J. Thorac. Cardiovasc. Surg.* 147 1604–1611. 10.1016/j.jtcvs.2013.09.050 24267779

[B39] Sato T. Watanabe A. Kondo H. Kanzaki M. Okubo K. (2015). Long-term results and predictors of survival after surgical resection of patients with lung cancer and interstitial lung diseases. *J. Thorac. Cardiovasc. Surg.* 149 70–71. 10.1016/j.jtcvs.2014.08.086 25439777

[B40] Sekine A. Satoh H. Baba T. Ikeda S. Okuda R. (2016). Safety and efficacy of S-1 in combination with carboplatin in non-small cell lung cancer patients with interstitial lung disease: A pilot study. *Cancer Chemother. Pharmacol.* 77 1245–1252. 10.1007/s00280-016-3040-8 27130459

[B41] Shah R. R. (2016). Tyrosine kinase Inhibitor-Induced interstitial lung disease: Clinical features, diagnostic challenges, and therapeutic dilemmas. *Drug Saf.* 39 1073–1091. 10.1007/s40264-016-0450-9 27534751

[B42] Shi L. Tang J. Tong L. Liu Z. (2014). Risk of interstitial lung disease with gefitinib and erlotinib in advanced non-small cell lung cancer: A systematic review and meta-analysis of clinical trials. *Lung Cancer* 83 231–239. 10.1016/j.lungcan.2013.11.016 24332320

[B43] Shimizu R. Fujimoto D. Kato R. Otoshi T. Kawamura T. (2014). The safety and efficacy of paclitaxel and carboplatin with or without bevacizumab for treating patients with advanced nonsquamous non-small cell lung cancer with interstitial lung disease. *Cancer Chemother. Pharmacol.* 74 1159–1166. 10.1007/s00280-014-2590-x 25245821

[B44] Shintani Y. Ohta M. Iwasaki T. Ikeda N. Tomita E. (2010). Predictive factors for postoperative acute exacerbation of interstitial pneumonia combined with lung cancer. *Gen. Thorac. Cardiovasc. Surg.* 58 182–185. 10.1007/s11748-009-0569-z 20401711

[B45] Simler N. R. Brenchley P. E. Horrocks A. W. Greaves S. M. Hasleton P. S. (2004). Angiogenic cytokines in patients with idiopathic interstitial pneumonia. *Thorax* 59 581–585. 10.1136/thx.2003.009860 15223865 PMC1747058

[B46] Suresh K. Voong K. R. Shankar B. Forde P. M. Ettinger D. S. (2018). Pneumonitis in Non-Small cell lung cancer patients receiving immune checkpoint immunotherapy: Incidence and risk factors. *J. Thorac. Oncol.* 13 1930–1939. 10.1016/j.jtho.2018.08.2035 30267842

[B47] Tomassetti S. Gurioli C. Ryu J. H. Decker P. A. Ravaglia C. (2015). The impact of lung cancer on survival of idiopathic pulmonary fibrosis. *Chest* 147 157–164. 10.1378/chest.14-0359 25166895

[B48] Tone M. Izumo T. Awano N. Kuse N. Inomata M. (2019). High mortality and poor treatment efficacy of immune checkpoint inhibitors in patients with severe grade checkpoint inhibitor pneumonitis in non-small cell lung cancer. *Thorac. Cancer* 10 2006–2012. 10.1111/1759-7714.13187 31482678 PMC6775002

[B49] Usui K. Tanai C. Tanaka Y. Noda H. Ishihara T. (2011). The prevalence of pulmonary fibrosis combined with emphysema in patients with lung cancer. *Respirology* 16 326–331. 10.1111/j.1440-1843.2010.01907.x 21114711

[B50] Watanabe N. Taniguchi H. Kondoh Y. Kimura T. Kataoka K. (2013). Efficacy of chemotherapy for advanced non-small cell lung cancer with idiopathic pulmonary fibrosis. *Respiration* 85 326–331. 10.1159/000342046 23171837

[B51] Watanabe S. Saeki K. Waseda Y. Murata A. Takato H. (2018). Lung cancer in connective tissue disease-associated interstitial lung disease: Clinical features and impact on outcomes. *J. Thoracic Dis.* 10 799–807. 10.21037/jtd.2017.12.134 29607151 PMC5864671

[B52] Weis S. M. Cheresh D. A. (2005). Pathophysiological consequences of VEGF-induced vascular permeability. *Nature* 437 497–504. 10.1038/nature03987 16177780

[B53] Xie Z. Zhou C. Qin Y. Ouyang M. Li S. (2019). [Diagnosis and treatment strategy for advanced severe lung cancer]. *Chin. J. Pract. Internal Med.* 39 416–419. 10.19538/j.nk2019050106

[B54] Yamaguchi S. Ohguri T. Ide S. Aoki T. Imada H. (2013). Stereotactic body radiotherapy for lung tumors in patients with subclinical interstitial lung disease: The potential risk of extensive radiation pneumonitis. *Lung Cancer* 82 260–265. 10.1016/j.lungcan.2013.08.024 24054547

[B55] Yoo H. Jeong B. H. Chung M. J. Lee K. S. Kwon O. J. (2019). Risk factors and clinical characteristics of lung cancer in idiopathic pulmonary fibrosis: A retrospective cohort study. *BMC Pulm. Med.* 19:149. 10.1186/s12890-019-0905-8 31412851 PMC6693185

