# Supplementary material for: Management and Prognosis of Interstitial Lung Disease With Lung Cancer (ILD-LC): A Real-World Cohort From Three Medical Centers in China
Source: Front Mol Biosci. 2021 Mar 31;8:660800. doi: 10.3389/fmolb.2021.660800 (PMC8044367; doi:10.3389/fmolb.2021.660800)
Supplement: Supplementary file 1 [file Table_1.DOCX]

Supplementary Material

**Supplementary Table 1.** Censored rate and median follow-up time of the ILD-LC cohort.

|  | All | Dead | Alive | Lost to follow-up | Censored | Censored Rate | Median follow-up time (Months) |
| --- | --- | --- | --- | --- | --- | --- | --- |
| All patients | 184 | 127 | 23 | 34 | 57 | 0.31 | 27 |
| Non-anticancer | 44 | 40 | 2 | 2 | 4 | 0.091 | Undefined |
| Early stage with surgery | 25 | 7 | 12 | 6 | 18 | 0.72 | 38.4 |
| Early stage with systematic therapy | 23 | 17 | 1 | 5 | 6 | 0.261 | 27 |
| Advanced stage with systematic therapy | 92 | 63 | 8 | 21 | 29 | 0.315 | 15.7 |

**Supplementary Table 2.** Subgroup Analysis of ILD-LC

|  | Censored | Dead | Median OS | p-value | HR | 95%CI |
| --- | --- | --- | --- | --- | --- | --- |
| Anti-cancer cohort | 53 | 87 | 11.1 | <0.0001 | 0.3014 | 0.1771 to 0.5130 |
| Non-anticancer cohort | 4 | 40 | 3.5 |  |  |  |
| Early stage with surgery | 18 | 7 | Undefined | <0.0001 | 0.2049 | 0.08764 to 0.4791 |
| Early-stage with systematic therapy | 6 | 17 | 14.2 |  |  |  |
| Advanced stage with systematic therapy | 29 | 63 | 7.2 | <0.0001 | 0.3581 | 0.2087 to 0.6147 |
| Advanced stage with non-anticancer | 2 | 34 | 3.2 |  |  |  |
| Systematic therapy with Anti-angiogenesis in Advanced LC | 7 | 15 | 9.3 | 0.0148 | 0.5226 | 0.3137 to 0.8705 |
| Systematic therapy with Anti-angiogenesis in Advanced LC | 22 | 48 | 6.1 |  |  |  |
